# Tuning the Mechanical and Adhesion Properties of Carbon Nanotubes Using Aligned Cellulose Wrap (Cellulose Nanotube): A Molecular Dynamics Study

**DOI:** 10.3390/nano10010154

**Published:** 2020-01-16

**Authors:** Mehdi Shishehbor, M. Reza Pouranian

**Affiliations:** School of Civil Engineering, Purdue University, West Lafayette, IN 47907, USA; mpourani@purdue.edu

**Keywords:** carbon nanotube, cellulose, molecular dynamics, adhesion, mechanical properties, nanopaper, toughness, strength

## Abstract

Improving the adhesion properties of carbon nanotubes (CNTs) at the molecular scale can significantly enhance dispersion of CNT fibers in polymer matrix and unleash the dormant extraordinary mechanical properties of CNTs in CNT-polymer nanocomposites. Inspired by the outstanding adhesion, dispersion, mechanical, and surface functionalization properties of crystalline nanocellulose (CNC), this paper studies the mechanical and adhesion properties of CNT wrapped by aligned cellulose chains around CNT using molecular dynamic simulations. The strength, elastic modulus, and toughness of CNT-cellulose fiber for different cellulose contents are obtained from tensile and compression tests. Additionally, the effect of adding cellulose on the surface energy, interfacial shear modulus, and strength is evaluated. The result shows that even adding a single layer cellulose wrap (≈55% content) significantly decreases the mechanical properties, however, it also dramatically enhances the adhesion energy, interfacial shear strength, and modulus. Adding more cellulose layers, subsequently, deceases and increases mechanical properties and adhesion properties, respectively. In addition, analysis of nanopapers of pristine CNT, pristine CNC, and CNT-wrapped cellulose reveals that CNT-wrapped cellulose nanopapers are strong, stiff, and tough, while for CNT and CNC either strength or toughness is compromised. This research shows that cellulose wraps provide CNT fibers with tunable mechanical properties and adhesion energy that could yield strong and tough materials due to the excellent mechanical properties of CNT and active surface and hydrogen bonding of cellulose.

## 1. Introduction

Engineering strong and tough materials has been the demand of many industries, such as automobile and aerospace, in the past decades. However, in most engineering materials, selecting one property is a compromise to the other. For example, typical strong engineering fibers such as carbon or glass fibers are brittle, whereas, most tough engineering polymers have low strength. To address both strength and toughness simultaneously requires advanced material design that incorporates strong and tough materials with energy dissipation strategies [1,2,3,4]. Carbon nanotube (CNT) with exceptional strength, stiffness, and toughness has been considered a promising material to achieve this goal [5]. However, agglomeration of CNTs due to their strong van der Waals interactions limits their solubility and dispersion and reduces both strength and toughness of the final product [6,7]. Currently the main established approach to address this problem is covalent or non-covalent surface modification of CNT with functional groups such as carboxyl groups or polymers such as Poly(3-alkylthiophenes) [6,8]. Although covalent surface modification is more stable, it changes the intrinsic mechanical properties of CNT [9,10]. For example, buckling behavior of O-CNTs (functionalization of CNTs with Oxygens and hydroxyl groups) with 6% oxygenation under compression load showed reduction in elastic modulus and failure strain of CNTs [11]. Additionally, Khoei and Khorrami (2016) reported reduction in the Young’s modulus, shear modulus, and ultimate stress of graphene-oxides (GO) by increasing the oxygenation [12]. Therefore, to retain intrinsic properties of CNT, non-covalent functionalization has been pursued by many researchers. For example, significant stress transfer and improvement in the mechanical properties of CNT-polymer nanocomposite were reported by wrapping poly(methyl methacrylate) (PMMA)-around CNT [13]. Experimental and molecular dynamics (MD) study of polyacrylonitrile (PAN)/CNT composite fibers revealed that (1) increasing alignment of PAN fibers significantly improves Young’s modulus and ultimate strength of the composite, and (2) increasing PAN-CNT interaction improves dispersion quality of CNTs [14,15].

Recent manufacturing of CNT/cellulose and graphene/cellulose nanocomposites for many applications such as porous and conductive textiles [16], flexible sensors [17], 3D printed conductive microfiber [18] and energy storage devices [16,19] shows cellulose as promising polymer for non-covalent functionalization. However, most studies on CNT/cellulose composite demonstrate reduction in strength by increasing CNT content (more than 5 wt%) due to CNT agglomeration and weak dispersion [20,21,22,23]. For instance, the mechanical properties of a highly conductive and light weight composite of CNT and cellulose nanofibrils (CNF) for different CNT/CNF weight ratios demonstrated reduction in tensile strength by increasing CNT/CNF ratio due to CNT agglomeration [20]. However, most recently, the fabrication of a multifunctional composite of CNFs/CNT with axially oriented regions of CNF networks, exhibits much higher strength (≈472.17 MPa) than previous works (6.47 times higher) due to the wrapped aligned nanocellulose network around CNT [23]. Observation of the results for both PAN-CNT [15] and CNF/CNT [23] shows that the non-covalently bonded aligned polymers on CNT surface improved the mechanical performance of the composite.

In addition to CNF, a new family of cellulose particles with nanoscale dimensions of 3–20 nm in width and 50–500 nm in length, called cellulose nanocrystals (CNCs), gained significant attention as a renewable, strong and stiff material [24,25,26,27]. For example, the elastic modulus of CNC in the axial direction is 110–200 GPa [24,28,29] and the reported values for the strength is in the range of 2–6 GPa [28,30,31]. CNCs can self-assemble into a liquid crystalline form and have highly active surface for functionalization [24,32]. The structure of CNC is very inspiring for forming a stable polymer wrap around CNT as all the cellulose chains in CNC are aligned and interacting through inter- and intra-hydrogen bonding.

In addition to experimental observations, the molecular dynamic (MD) method has been extensively used in the past for mechanical properties of CNT [11,33], CNC [24,29,31,34,35,36,37], polymer wrapped CNT [38,39,40,41], and CNT-polymer nanocomposite [42,43,44]. For example, Yu et al. employed MD simulations to show improvement in the interfacial energy of CNT-polymer due to aromatic polymer chains [43]. MD simulations for sliding of two CNF and two CNT in direct contact showed significantly higher surface energy of CNF than CNC due to hydrogen bonding [32]. Additionally, recent theoretical studies on nanotube of cellulose chains, shows the possibility of forming a stable and even self-assembled nanotube of cellulose through hydrogen bonding [45,46]. The authors mentioned the possible self-assembly of these chains as single walled or multiwalled nanotube through solvation, particularly in benzene and its derivatives [46].

Therefore, due to (1) high mechanical properties and high surface energy of cellulose, (2) positive previous results in the literature on the effect of aligned polymer chains on CNT surface, (3) inspired by the structure of CNC, where aligned cellulose chains are packed together through inter- and intra-hydrogen bonding, and (4) theoretical study on the stability and self-assembly of cellulose nanotube; wrapping single walled and multiwalled cellulose nanotube (aligned cellulose fibers in circular form) around CNT could result in stable fibers with much better functionality, and tunable mechanical properties that will be evaluated in this paper by MD simulations.

Finally, we use the obtained results from MD simulations for theoretical evaluation of strength and stiffness of staggered CNT-wrapped cellulose nanopaper. For staggered structures, sliding of the fibers (tablets) is essential for providing high stiffness, strength, and toughness and therefore the ratio of fiber strength and stiffness to interface strength is a crucial parameter [47,48]. For example, pristine CNT has very high strength (≈100 GPa) and stiffness (≈1000 GPa), but very low interfacial strength, and therefore pristine CNT nanopaper has low strength but high toughness. On the other hand, CNC has much higher interfacial strength than CNT, but much lower strength (2–6 GPa) and stiffness (110–200 GPa). As previous study shows [31], the high surface energy of CNC leads to catastrophic brittle failure of staggered CNC nanopapers when the strength of nanopaper reaches the ultimate strength of CNC (2–6 GPa) and, therefore, the toughness of nanopaper is compromised for its strength and stiffness (CNC breaks instead of sliding). Therefore, we surmise that CNT-wrapped cellulose fibers that incorporate high strength and stiffness of CNT with high surface energy of CNC could yield strong, stiff, and tough nanopapers. The aim of this paper is to evaluate (1) the effect of adding aligned cellulose wrap around CNT on the mechanical and adhesion properties of CNT and (2) how these properties affect the strength, stiffness, and toughness of nanomaterials made by CNT-wrapped cellulose (for example, a nanopaper of CNT-wrapped cellulose). 

## 2. Materials and Methods

In this section, the model preparation and molecular dynamic simulation setup for calculating mechanical properties and surface energy is explained.

### 2.1. CNC and CNT Wrapped by Cellulose

**Figure 1:**
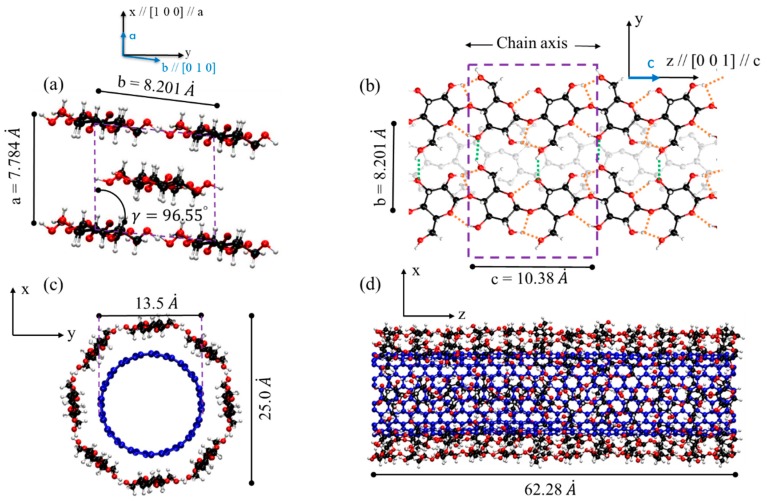
Molecular model of crystalline nanocellulose (CNC), and carbon nanotube (CNT) wrapped by a single layer of cellulose chains. Red spheres represent oxygen atoms, black spheres represent carbon atoms in CNC, white spheres represent hydrogen atoms, and blue spheres are carbon atoms in CNT. (**a**) Atomistic structure of CNC unit cell from cross-section view adapted from [29,49]. (Reproduced with permission from [29]. Elsevier, 2018). (**b**) CNC view along chain direction (c-axis). (**c**) Cross-section view of CNT-wrapped by one layer of aligned cellulose chains (similar to CNC). (**d**) View of CNT-wrapped cellulose along the axial direction of CNT.

CNC is formed by stacking of highly ordered cellulose chains (linear chain of 1–4 linked β-D glucopyranose) during biosynthesis process. The most stable form of CNC is Cellulose Iβ, found mostly in plants, with experimentally measured unit cell values of the following: *a* = 7.784 *Å*, *b* = 8.201 *Å*, *c* = 10.380 *Å*, *α* = 90°, *β* = 90°, *γ* = 96.55° at room temperature (Figure 1a) [49]. The stability of the structure is mainly supported by inter- and intra-chain hydrogen bonding as shown with green and orange dashed lines in Figure 1b and the highest stiffness and strength is in the axial direction (c-direction) due to covalent bonding. Similarly, when the cellulose chains are aligned and wrapped around CNT with inter- and intra-hydrogen bonding between chains, the wrapped is very stable and the stiffness and the strength of cellulose chains are fully engaged in the axial direction as shown in Figure 1c,d, respectively. The cross-section view and the view along axial direction for the molecular model of CNT-wrapped by aligned cellulose chains are shown in Figure 1c,d, respectively. The number of cellulose chains in each layer is calculated based on the unit cell parameters of CNC, i.e., *a* and *b*. The length of the CNT-wrapped is designated by the number of cellulose unit cells in the axial direction (*c* = 10.38 Å) and both CNT and cellulose have similar length.

### 2.2. Single and Multilayer Cellulose Wrap

**Figure 2:**
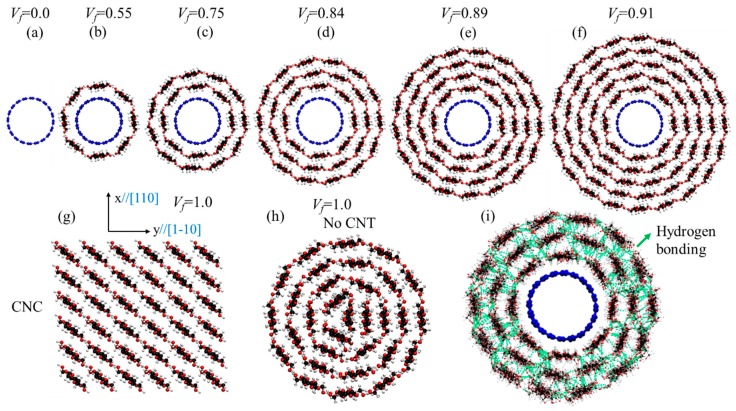
Molecular model of CNT-wrapped with multi-layer cellulose chains at different cellulose content (*V_f_*). (**a**) *V_f_* = 0%, only CNT (10,10) (**b**) *V_f_* = 55%, CNT-wrapped with one layer of cellulose. (**c**) *V_f_* = 75%, CNT-wrapped with two layers of cellulose. (**d**) *V_f_* = 84%, CNT-wrapped with three layers of cellulose. (**e**) *V_f_* = 89%, CNT-wrapped with four layers of cellulose. (**f**) *V_f_* = 91%, CNT-wrapped with five layers of cellulose. (**g**) *V_f_* = 100%, CNC with 36 chains and [110] and [1–10] surfaces. (**h**) *V_f_* = 100%, cellulose wrapped with no CNT. (**i**) Hydrogen bonding between chains in multiwalled cellulose nanotube is shown with green dashed lines for *V_f_* = 84% after equilibration.

The number of cellulose layers on CNT could affect both mechanical and surface energy. More layers add more cellulose content (*V_f_*) and since cellulose mechanical properties is lower than CNT, it could reduce the mechanical performance of the bundle. On the other hand, the higher the number of layers, the more active surface area is participating in the composite. Therefore, the effect of the number of cellulose layers (*V_f_*) on the mechanical and surface energy is investigated. Figure 2 represents the molecular model for different *V_f_* of cellulose, CNC (*V_f_* = 100%) and a multilayer cellulose wrap with no CNT (*V_f_* = 100%).

The cellulose content that is studied here by adding cellulose layers varies from 0.0% for pure CNT (Figure 2a), to 55% for one layer cellulose (Figure 2b), 75% for two layers of cellulose (Figure 2c), 84% for three layers of cellulose (Figure 2d), 89% for four layers of cellulose (Figure 2e), and 91% for five layers of cellulose (Figure 2f). For comparison of the results with the cases where there only cellulose exists (100% cellulose), two case studies of CNC (Figure 2g) and cellulose wrap with no CNT (Figure 2h) are studied here. For CNC, the diamond shape structure with 36 chains, [110] and [1–10] surfaces as the most recommended structure model for CNC is studied here [24,31]. According to previous theoretical study, increasing the diameter of the single-walled cellulose nanotubes results in a more stable structure due to more stable inter-molecular hydrogen bonds [46]. For multiwalled cellulose nanotubes, the structure is even more stable as there is inter-walled hydrogen bonding between cellulose chains (as shown with green lines in Figure 2i) similar to those that exist in CNC. It is worth mentioning that in many previous studies the CNT was used in the bulk cellulose, but there are experimental results on strong and highly conductive CNT/cellulose fiber [18]. For example, Li et al. reported strong and highly conductive microfibers of CNT/cellulose for 3D printing applications [18].

### 2.3. Mechanical and Surface Energy Tests

**Figure 3:**
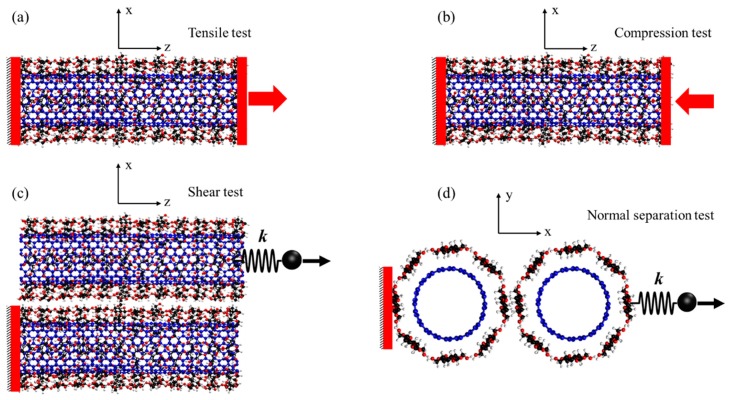
Mechanical and surface energy tests for calculating mechanical performance, shear force, and adhesion energy of fibers. (**a**) Tensile test, (**b**) compression test, (**c**) shear test, and (**d**) normal test for adhesion energy.

For evaluating the mechanical properties of the CNT-wrapped by aligned cellulose, two mechanical tests (i.e., tensile test and compression test) were performed. For both tests, the boundary atoms at one side were fixed, while displacement was applied (at the speed of 0.05 Å/ps) to the opposite side boundary atoms in the direction of tension (Figure 3a) or compression (Figure 3b) for tensile and compression tests, respectively. The stress was calculated by summing all reaction forces and dividing them over the cross-section area. The strain is engineering strain that is obtained by dividing change in length over the initial length of the specimen. The stress-strain curves for tensile and compression tests were obtained for all case studies shown in Figure 2 and were compared against each other. For calculating the shear force and adhesion energy between CNT-wrapped cellulose fibers, two separation tests were performed in shear (Figure 3c) and normal (Figure 3d) directions, respectively. For both shear and normal tests, a steering force was applied to the top fiber (*x* direction) in the direction of shear (*z* direction) and normal (*x* direction), respectively, while boundary atoms of the bottom fiber were fixed.

The adhesion energy then can be calculated based on the following equations [44]:

(1) $$\Delta E=E_{total}-\left(E_{1}+E_{2}\right),$$

(2) $$\gamma =\frac{\Delta E}{2A},$$

where $E_{total}$ is the total energy of the system including two fibers and the interaction energy between them, $E_{1}$ and $E_{2}$ are the total energy of isolated fiber one and fiber two (when they are at infinity), respectively, ΔE is the interaction energy between two fibers, γ is the adhesion energy, and A is the effective area between two fibers obtained by multiplying the length and the diameter of the fiber.

### 2.4. Molecular Dynamics Procedure

**Figure 4:**
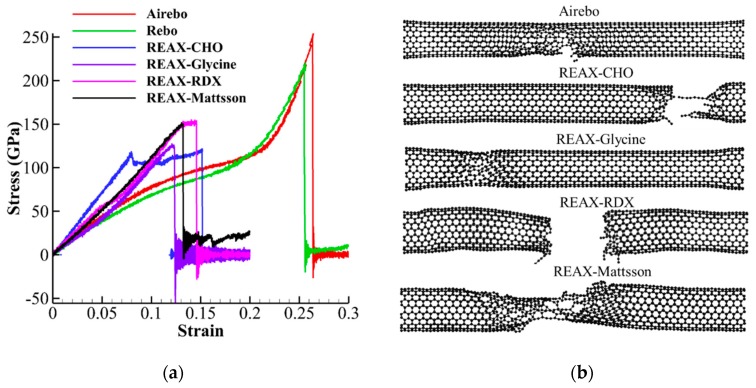
Stress-strain curves and fracture of CNT for different REAXFF parameters, Airebo and Rebo forcefield. (**a**) Stress-strain curves. (**b**) Fracture of CNT for different force fields.

All the simulations were performed using LAMMPS [50] MD package and REAXFF Forcefield [51,52] and timestep of 0.5 fs. For all the simulations, first, the system was minimized using conjugate gradient (CG) and Hessian-free truncated Newton (HFTN) methods, and then was equilibrated at 300 *K* temperature for 500 ps in NVT ensemble using Nosé-Hoover thermostat. Using REAXFF force fields for simulations has advantages of (1) dynamic bond breaking and bond formation between atoms based on bond order concept, (2) dynamic charge equilibration and atom charge assignment at each time step, and (3) accuracy in order of quantum mechanical calculations. However, REAXFF is, computationally, a very expensive force field and the timestep usually used for simulations is less than 1 fs. During the past decade, many REAXFF parameters have been developed for different materials and environment [52,53,54,55]. As carbon is the only atom involved in CNT, many RAXFF sets of parameters can be used for CNT, but not all of them could be suitable for its mechanical properties. On the other hand, for cellulose, three atoms of carbon, oxygen, and hydrogen are involved that are more limiting than CNT. Previous study on the elastic modulus and strength of CNC shows that the RAXFF parameters that were developed by Mattsson et al. [56] for simulation of shocked polymers are suitable for CNC [34]. However, these parameters need to be tested for CNT before using it for CNT-CNC composite. Aa a result, we tested four different RAXFF parameters and two other popular bond order force fields for CNT, i.e., Rebo [57] and Airebo [58], for capturing the mechanical properties of the CNT. Figure 4 represents the stress-strain curves and fractured specimens from tensile tests of CNT (10,10) using Rebo, Airebo, REAXFF-CHO [59], REAXFF-Glycine [60], REAXFF-RDX [61], and REAXFF-Mattsson [56]. The result shows that the Young’s modulus of all REAXFF force fields, Rebo and Airebo, are in the same range 900–1100 GPa and consistent with numerical and experimental results [33], except RAXFF-CHO that shows higher Young’s modulus (1350 GPa). The strength values for RAXFF force fields are in the range of 100–150 GPa lower than those from Rebo and Airebo (220–250 GPA). However, the strength and failure strain from REAXFF force fields are more consistent with those from tight bonding and density function theory calculations (strength of 110 GPa and failure strain of 0.2). Additionally, the fractured model under tensile load displays more ductile failure (tilt failure surface) in Airebo, Rebo (similar to Airebo), REAXFF-CHO, and REAXFF-Mattsson, while more brittle failure is observed in RAXFF-RDX and RAXFF-Glycine as shown in Figure 4b. For small diameter CNT (chiral indices less than 14), previous results indicates that ductile fracture is prominent [62]. Therefore, it can be concluded that REAXFF-Mattsson that is previously shown to be suitable for cellulose [34], is also appropriate for modeling the mechanical properties of CNT and therefore was used for all of our calculations for CNT-wrapped cellulose in this paper.

## 3. Results and Discussion

In this section the results for tensile, compression, normal separation (adhesion energy), and interfacial shear tests are shown and discussed.

### 3.1. Tensile Properties of CNT-Wrapped Aligned Cellulose

**Figure 5:**
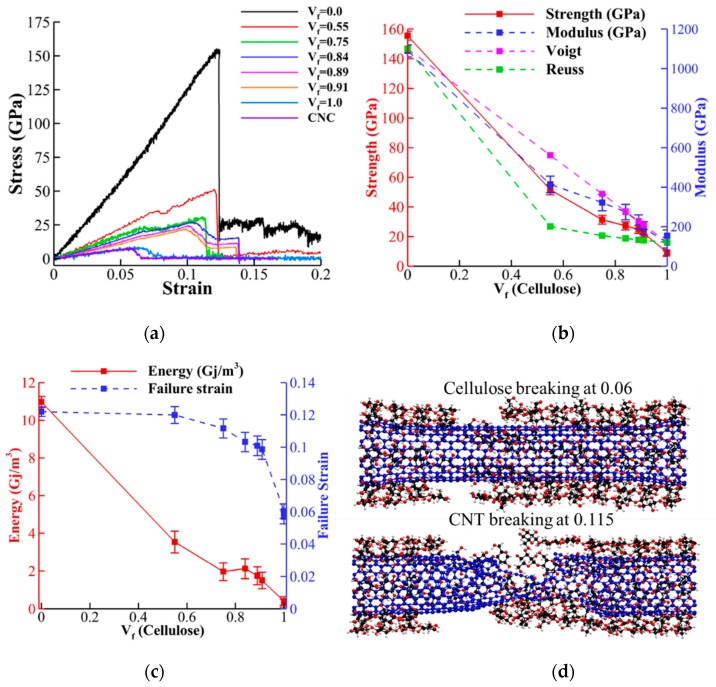
Mechanical properties of CNT-wrapped cellulose at different cellulose contents. (**a**) Represents stress-strain curves. (**b**) Variation of strength and Young’s modulus for different cellulose content is shown. (**c**) Variation of toughness and failure strain for different cellulose content is shown. (**d**) Fractured model for single walled cellulose (*Vf* = 55%) shows cellulose chains breaking at 6% strain and then nanotube fails at 0.125 strain.

After performing tensile tests (procedure explained in Section 2.3), the stress-strain curves, strength (maximum stress), Young’s modulus (slope of stress-strain), failure strain (strain associated with strength) and toughness (area below stress-strain curves) for different *V_f_* were compared (Figure 5). Figure 5a, demonstrates the stress-strain curves for CNT-wrapped aligned cellulose with different *V_f_* (Figure 2a–h) and also for CNC (Figure 2g). The strength and Young’s modulus extracted from these curves are shown in Figure 5b with red solid and blue dashed curves, respectively. The values of strength vary almost linearly from 150 GPa for CNT (*V_f_* = 0.0) to 6.0 GPa for CNC (*V_f_* = 1.0). In addition, comparison of the results between CNC and cellulose wrapped with no CNT (Figure 2h) shows negligible difference between their strength values (7.0 GPa for cellulose wrapped with no CNT versus 6.0 GPa for CNC). The Young’s modulus values (blue dashed curve in Figure 5b) changes from 1100 GPa for CNT (*V_f_* = 0.0) to 140.0 GPa for CNC (*V_f_* = 1.0) with negligible difference between CNC and cellulose wrapped with no CNT (140.0 GPa for CNC and 125 GPa for cellulose wrapped with no CNT).

Here, we also compared the elastic modulus with theoretical upper bound (Voigt model) and lower bound (Reuss model) values from composite materials context [63]. In the Voigt model the equivalent elastic modulus can be obtained as follows:

(3) $$E_{c}=E_{Celulose}V_{f}+E_{CNT}(1-V_{f}).$$

In the Reuss model, the inverse rule of mixture is used for representing the lower bound as follows:

(4) $$E_{c}=E_{Celulose}V_{f}+E_{CNT}(1-V_{f}),$$

where in both equations, *E_c_* is the elastic modulus of composite, $E_{Celulose}$ is the elastic modulus of cellulose and $E_{CNT}$ is the elastic modulus of CNT. The values for Voigt and Reuss models (shown with pink and green dashed lines in Figure 5b, respectively) indicates that for high cellulose content (*V_f_* ≥ 0.84), the Voigt model and numerical values have less than 5% difference. For *V_f_* = 0.75 and *V_f_* = 0.55, the Voigt prediction is 15% and 35% higher than the numerical values. Variation of toughness and failure strains with respect to *V_f_* are shown in Figure 5c with red solid and blue dashed lines, respectively. The results for toughness almost linearly decrease with increasing *V_f_* (similar to strength and elastic modulus) from 11 Gj/m^3^ for CNT to 0.35 Gj/m^3^ for CNC. By increasing the cellulose content, the failure strain slowly decreases from the failure strain of CNT (0.12) to the average value of CNC (0.06) and CNT failure strains (0.09).

**Figure 6:**
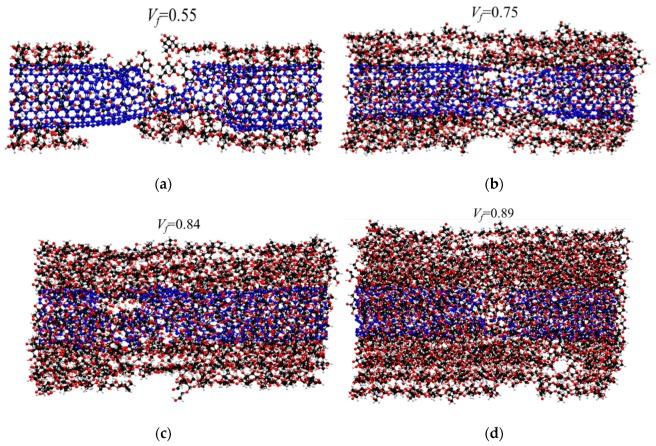
Fractured CNT-wrapped cellulose at different cellulose content and transition from more ductile fracture to more brittle by increasing cellulose content. (**a**) Ductile fracture of CNT at *V_f_* = 55%. (**b**) *V_f_* = 75% (**c**) *V_f_* = 84% and (**d**) Brittle fracture of CNT at *V_f_* = 89%.

The failure mechanism for *V_f_* = 0.55 (shown in Figure 5d) demonstrates breaking of cellulose chains at 0.06 strain, causing reduction in load bearing (shown with local reduction in stress in Figure 5a right after 0.06 strain). However, the ultimate failure happens by breaking of CNT at 0.115 (shown in Figure 5b). Additionally, the result shows that, by increasing the cellulose content, the inter-chain hydrogen bonds between cellulose chains induces more and more pressure on CNT and, as it is shown previously [64], increasing lateral pressure on CNT reduces the failure strain of CNT significantly. The failed specimens for *V_f_* = 0.55, 0.75, 0.84, and 0.89, as shown in Figure 6a–d, respectively, display transition failure of CNT from more ductile failure (tilted failed cross-section) to more brittle (straight failed cross section) [62]. We also studied the effect of length on the mechanical properties and failure mechanism for *V_f_* = 0.55, and the results do not show significant differences (as shown in Appendix A).

### 3.2. Compression Properties of CNT-Wrapped Cellulose

**Figure 7:**
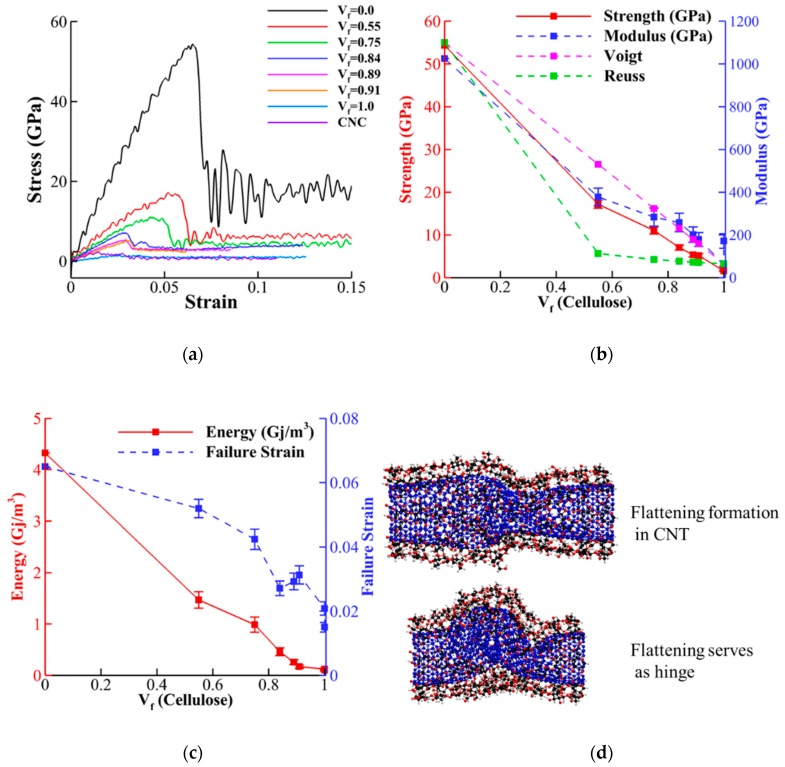
Compressive properties of CNT-wrapped cellulose at different cellulose content. (**a**) Stress-strain curves. (**b**) Variation of strength and Young’s modulus for different cellulose content is shown. (**c**) Variation of toughness and failure strain for different cellulose content is shown. (**d**) Buckling for CNT-wrapped single walled cellulose (*V_f_* = 55%) shows flattening and hinge formation in CNT similar to those observed in pristine CNT [65].

Figure 7 illustrates the stress-strain curves, strength (maximum compressive stress), Young’s modulus (slope of stress-strain), failure strain (strain associated with strength), and toughness (area below stress-strain curves) of CNT-wrapped cellulose for different *V_f_* under compressive loading. The strength and Young’s modulus extracted from stress-strain curves (Figure 7a) are shown in Figure 5b with red solid and blue dashed curves, respectively. Similar to tensile properties, the values of strength vary almost linearly from 55 GPa for CNT (*V_f_* = 0.0) to 2.5 GPa for CNC (*V_f_* = 1.0). Comparison of the results between CNC and cellulose wrapped with no CNT (Figure 2h) shows negligible difference between their strength values (2.0 GPa for cellulose wrapped with no CNT versus 2.5 GPa for CNC). Figure 7b shows that the Young’s modulus values (blue dashed line) varies from 1100 GPa for CNT (*V_f_* = 0.0) to 75.0 GPa for CNC (*V_f_* = 1.0) with negligible difference between CNC and cellulose wrapped with no CNT (75.0 GPa for CNC and 50 GPa for cellulose wrapped with no CNT). Similar to tensile tests, here, we also compared the elastic modulus with theoretical upper bound (Equation (3) for Voigt model) and lower bound (Equation (4) for Reuss model). The values for Voigt and Reuss models (shown with pink and green dashed lines in Figure 7b, respectively) indicates that for high cellulose content (*V_f_* ≥ 0.84), the Voigt model predicts the numerical values with less than 7% error. For *V_f_* ≥ 0.75 and *V_f_* = 0.55, however, the Voigt values are 15% and 45% higher than the numerical values. The results for toughness (red solid line in Figure 7c) almost linearly decreases with increasing *V_f_* from 4.2 Gj/m^3^ for CNT to 0.125 Gj/m^3^ for CNC. The variation of failure strain (blue dashed line in Figure 7c) shows that increasing the cellulose content decreases failure strain rapidly from the failure strain of CNT (0.06) to the value of CNC (0.02). The buckling mechanism observed for *V_f_* = 0.55 (shown in Figure 7d) is similar to buckling of CNT reported previously [65,66,67]. This can be explained by negligible compressive strength and stiffness of cellulose chains compared to CNT.

**Figure 8:**
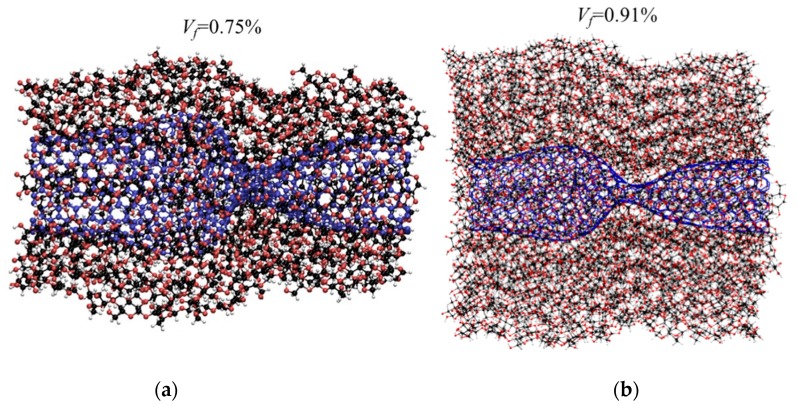
Buckling of CNT-wrapped cellulose during compression tests shows flattening of CNT. (**a**) *V_f_* = 75% and (**b**) *V_f_* = 91%.

The buckled cases for CNT-wrapped cellulose with *V_f_* = 0.75 and 0.91 (shown in Figure 8a,b, respectively) shows similar flattening regardless of cellulose content. Additionally, the effect of length on the mechanical properties and failure mechanism in compression test for *V_f_* = 0.55 shows 40% drop in strength and Young modulus and 70% drop in toughness as the length changes from 6 to 24 nm due to formation of second and third flattening in buckling (as shown in Appendix A).

### 3.3. Adhesion Energy

**Figure 9:**
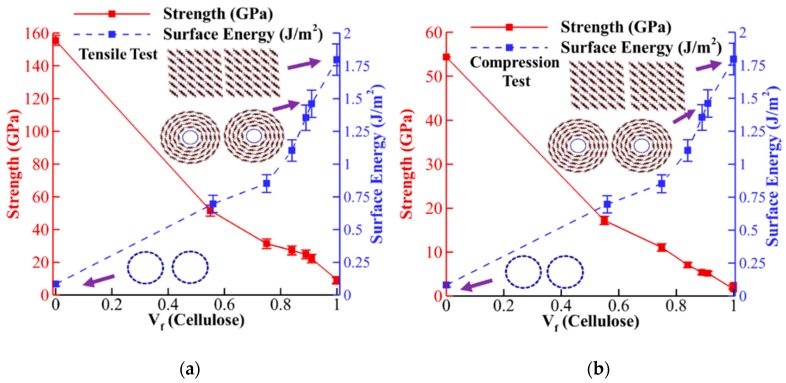
The variation of surface energy is compared with the strength and Young’s modulus by increasing cellulose content for the (**a**) tensile test and (**b**) compression test.

The values of surface energy for different cellulose content are shown in Figure 9 with blue dashed lines. For comparison, the variation of surface energy and strength for tensile and compression tests are plotted together in Figure 9a,b, respectively. The surface energy for CNT (*V_f_* = 0.0) is 0.08 J/m^2^ (0.48 nN per unit length) which is in the range of previous reported experimental values (0.36 per unit length) [68]. For CNC (*V_f_* = 1.0), the obtained value for adhesion energy (1.76 J/m^2^) is in the range of previously reported value [69] and an order of magnitude higher than CNT due to hydrogen bonding [32]. The result shows that for CNT-wrapped cellulose, the surface energy increases monstrously by increasing cellulose content from 0.72 J/m^2^ for *V_f_* = 0.55 to 1.4 J/m^2^ for *V_f_* = 0.91 (Figure 9). Comparing the trend of tensile strength and surface energy shows that, although adding one layer of cellulose wraps (*V_f_* = 0.55) could significantly reduce strength, it also significantly increases surface energy. For example, although the strength drops by 65% from 150 to 52 GPa, the surface energy increases by 900% from 0.08 to 0.72 J/m^2^ (Figure 9a). Similar comparison is also observed in compression test where strength drops by 67% from 55 to 18 GPa, while, the surface energy increases by 900% from 0.08 to 0.72 J/m^2^ (Figure 9b).

### 3.4. Interfacial Shear Strength

**Figure 10:**
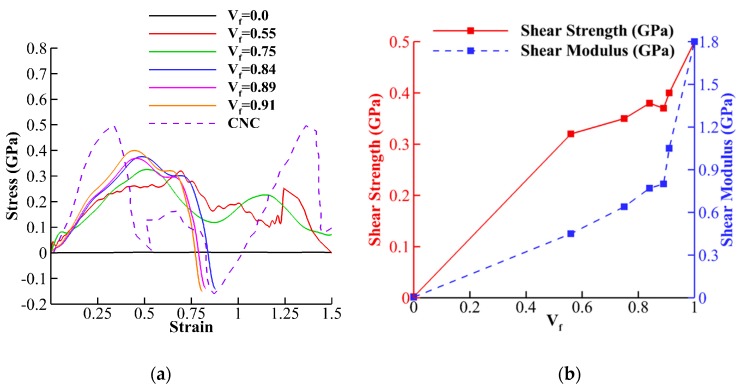
(**a**) Shear stress-strain curves for stress transfer between CNT-wrapped cellulose and (**b**) extracted strength and shear modulus from stress-strain curves are shown.

After simulations of shear tests between CNT-wrapped cellulose fibers for different cellulose content (Figure 3c), the shear stress-strain curves, strength, and shear modulus are obtained. The stress-strain curves show that for CNT (*V_f_* = 0.0), shown with a black solid line in Figure 10a, the value shear stress transfer is insignificant with respect to CNC (purple dashed line in Figure 10a). By increasing the cellulose content to *V_f_* = 0.55 (one layer cellulose wrap), and due to hydrogen bonding, the shear transfer significantly increases. Figure 10b compared the shear strength and shear modulus values extracted from Figure 10a for different *V_f_*. The shear strength varies from insignificant value of 0.002 GPa for CNT-CNT interface to 0.5 GPa for CNC-CNC interface. For *V_f_* = 0.55, the shear strength is 0.33 GPa, while adding more layers slowly increases the strength to 0.39 GPa for *V_f_* = 0.91. The shear modulus (dashed blue line in Figure 10b) almost linearly increases from 0.004 GPa for *V_f_* = 0.0 to 1.05 GPa for *V_f_* = 0.91. The results indicate that after one layer cellulose wrap (*V_f_* = 0.55), adding more cellulose layers only has a significant effect on the increases of shear modulus, while there is only slight improvement in the shear strength (Figure 10b).

### 3.5. Nanopaper of CNT-Wrapped Cellulose

**Figure 11:**
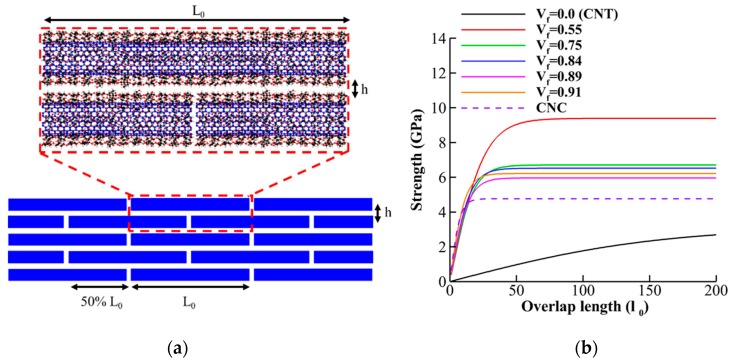

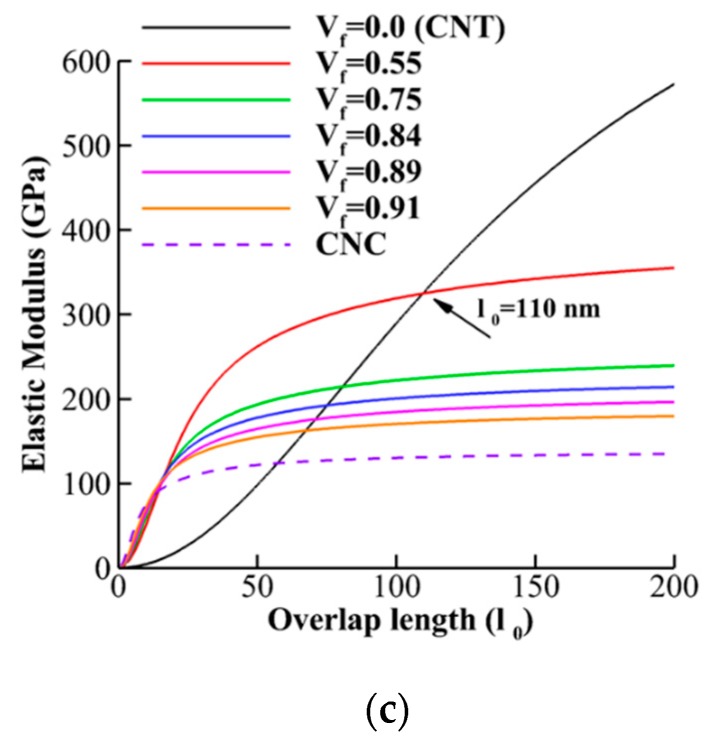
(**a**) Staggered arrangement of CNT-wrapped cellulose fibers with 50% overlap length. (**b**) Strength of nanopaper for different overlap length and cellulose content (**c**).

Here we use the results obtained in prior sections for theoretical evaluation of optimum design of nanopapers from CNT-wrapped cellulose (shown in Figure 11a). Previous studies on the microstructure of some biological materials such as nacre, bone, and teeth suggested that staggered (brick-and-mortar) arrangement of high-aspect ratio fibers (shown in Figure 11a) increases stiffness, strength, and toughness simultaneously [1,70]. In staggered arrangement design, in addition to interfacial properties between fibers and mechanical properties of the fiber, the value of overlap length for stress transfer between fibers play a crucial role [47,48]. Previous studies revealed that 50% overlap length in an optimum design maximizing the mechanical properties such as stiffness and strength [71]. According to shear-lag-model analytical relationships for strength and elastic modulus based on continuum shear-lag model have been proposed [72]:

(5) $$E=\frac{E_{c}}{1+2[(1+\cosh(l_{0}/l))/\sinh(l_{0}/l)](l/l_{0})},$$

(6) $$\sigma =\frac{\sinh(l_{0}/l)\gamma^{cr}E_{c}h_{c}}{2l[1+\cosh(l_{0}/l)]},$$

where $l=\sqrt{\frac{E_{c}h_{c}^{2}}{4G}}$ is length scale for stress transfer between fibers, G is the shear modulus of the interface, $E_{c}$ is the Young’s modulus of an individual fiber, $h_{c}$ is the inter-layer thickness, $l_{0}$ is the overlap length, and $\gamma^{cr}$ is the critical interlayer shear strain. Here, we use the results obtained from tensile tests and shear tests to feed the parameters in the analytical equations (Equations (5) and (6)). Figure 11b demonstrates the variation of the strength in nanopapers of CNT-wrapped cellulose with different overlap length and cellulose content. The result indicates that CNT with 55% cellulose wrap content (*V_f_* = 0.55) has the highest saturated strength (9.5 GPa) and pristine CNT (*V_f_* = 0.0) has the lowest saturated strength (3 GPa). For 0.75 ≤ *V_f_* ≤ 0.91 the values of the saturated strength are in the close range of 5.8–6.5 GPa and for CNC, the saturated strength is around 5 GPa. As previous study shows [31] the high surface energy of CNC leads to catastrophic brittle failure of nanopaper (CNC breaks instead of sliding) as the saturated strength after 30 nm overlap length (≈5 GPa) is higher than the strength of CNC (3–6 GPa) and, therefore, the toughness of nanopaper is compromised regarding its strength and stiffness. With CNT-wrapped cellulose, however, the ultimate strength (52 GPa for *V_f_* = 0.55 and 20–40 GPa for 0.75 ≤ *V_f_* ≤ 0.91) is much higher than the saturated strength (9.5 GPa for and 5.8–6.5 GPa for 0.75 ≤ *V_f_* ≤ 0.91) and therefore fiber sliding takes place instead of fiber breaking and brittle failure. For *V_f_* = 0.55, saturated strength takes place at *l*_0_ = 75 nm (9.5 GPa), while for 0.75 ≤ *V_f_* ≤ 0.91, saturated strength happens around *l*_0_ = 50 nm. Finally, for CNC, *l*_0_ = 30 nm is the overlap length for saturation of strength (≈5.0 GPa). Figure 11b shows the change in the elastic modulus of nanopaper as overlap and cellulose content varies. The result shows that the elastic modulus of nanopaper of pristine CNT varies significantly with length and for *l*_0_ ≤ 60 nm, it has the lowest value among other case studies (140 GPa). Then, in the range of 60 nm ≤ *l*_0_ ≤ 110 nm, the modulus varies from the modulus of CNC, 140 GPa, to 330 GPa for modulus of CNT-with one layer of cellulose (*V_f_* = 0.55) at *l*_0_ = 110 nm. For *V_f_* = 0.55, 80% of saturated modulus (400 GPa) takes place at *l*_0_ = 100 nm (320 GPa), while for 0.75 ≤ *V_f_* ≤ 0.91, 80% saturated modulus happened around *l*_0_ = 75 nm. Finally, for CNC, *l*_0_ = 30 nm is the overlap length for 80% saturation of modulus (112 GPa).

## 4. Summary and Conclusions

In this paper, the mechanical and adhesion properties of CNT wrapped by single walled and multiwalled cellulose were evaluated via molecular dynamic simulations and then utilized in an analytical solution to show potential application of the results in designing CNT-wrapped cellulose nanopapers. The strength, elastic modulus, and toughness of CNT wrapped cellulose for different cellulose content are obtained from tensile and compression tests. For tensile test, the values of strength, Young’s modulus, and toughness with respect to cellulose content varies almost linearly from CNT values (*V_f_* = 0.0) to CNC values (*V_f_* = 1.0). For example, the Young’s modulus values almost linearly change from 1100 GPa for CNT (*V_f_* = 0.0) to 140.0 GPa for CNC (*V_f_* = 1.0). For low cellulose content, the tensile failure happens in cellulose and causes reduction in load bearing, but the ultimate failure takes place by breaking CNT. By increasing the cellulose content, however, the inter-chain hydrogen bonds between cellulose chains induces lateral pressure on CNT and reduces the failure strain of CNT (CNT becomes more brittle). Similar to tensile properties, in compression tests, the values of strength, Young’s modulus, and toughness with respect to cellulose content varies almost linearly from CNT values (*V_f_* = 0.0) to CNC values (*V_f_* = 1.0). The buckling mechanism observed for CNT-wrapped cellulose is similar to buckling of CNT due to negligible compressive strength and stiffness of cellulose chains compared to CNT.

For adhesion tests, the effect of cellulose content on surface energy, interfacial shear strength, and shear modulus were evaluated. The result from surface energy shows that for CNT-wrapped cellulose, the surface energy is significantly higher than CNT and increases monstrously by increasing cellulose content from 0.72 J/m^2^ for *V_f_* = 0.55 to 1.4 J/m^2^ for *V_f_* = 0.91. In addition, comparing the trend of tensile strength and surface energy shows that, although adding one layer of cellulose wraps (*V_f_* = 0.55) could significantly reduce the strength of fiber, but it also significantly increases surface energy. For interfacial shear test, the results show that, due to hydrogen bonding, even one layer cellulose wrap significantly increases the shear transfer between fibers. However, after the first layer, adding more cellulose content shows more improvement on the increases of shear modulus than on shear strength.

Finally, the mechanical properties of the nanopaper of CNT-wrapped cellulose shows that CNT with 55% cellulose wrap content (*V_f_* = 0.55) has the highest saturated strength (9.5 GPa), pristine CNT (*V_f_* = 0.0) has the lowest saturated strength (3 GPa), and for CNC, the saturated strength is around 5 GPa. This indicates that for pristine CNT, the nanopaper has low strength but high toughness (sliding of the fibers are the failure mechanism since the strength is much lower than ultimate strength of CNT). For CNC, on the other hand, the interfacial strength is much higher than CNT, and saturated strength is close to ultimate strength of CNC (2–6 GPa) and, therefore, the toughness of nanopaper is compromised for its strength and stiffness (CNC breaks instead of sliding). With CNT-wrapped cellulose, however, the ultimate strength (52 GPa for *V_f_* = 0.55 and 20–40 GPa for 0.75 ≤ *V_f_* ≤ 0.91) is much higher than the saturated strength (9.5 GPa for and 5.8–6.5 GPa for 0.75 ≤ *V_f_* ≤ 0.91) and therefore their nanopaper is strong, stiff, and tough (fiber sliding is the failure mechanism).

Although we only discussed one potential application of CNT-wrapped with cellulose here, they could also be used for many others such twisted rope, bundles, or dispersed as reinforcement agent in polymer matrices due their highly active surface.

Additionally, although in most experimental studies, it has been shown that cellulose/CNT are a good combination for electrical conductivity [17,18,20], this study was focused on the mechanical performance; and the electronic properties should be evaluated in a separate study. This study evaluated the effect of different cellulose content on CNT/cellulose fiber performance and serves as a road map for tuning different mechanical and adhesion properties of CNT/cellulose based on cellulose content. Our result for nanopaper shows that even using a very high volume content of aligned cellulose (could represent a bulk model) would produce strong, stiff, and tough nanopaper. In addition, although this study was focused on CNT/cellulose fiber, the high volume cellulose content case study could be a good representation of the bulk model as both mechanical and adhesion properties result converge to pure CNC.

## Figures and Tables

**Figure A1:**
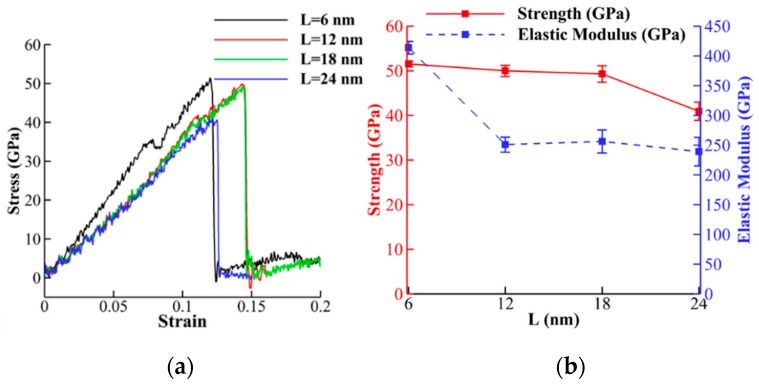

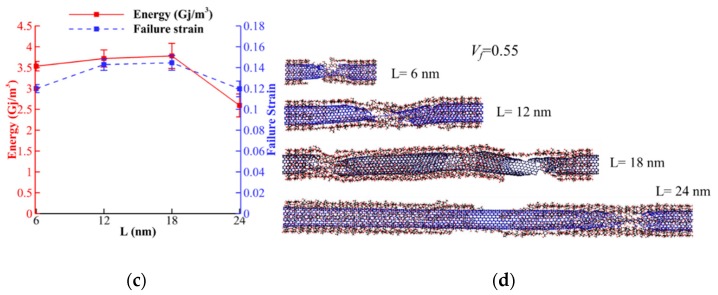
Effect of length on the tensile properties of CNT-wrapped cellulose with *V_f_* = 0.55. (**a**) Stress-strain curves. (**b**) Variation of strength and Young’s modulus for length. (**c**) Variation of toughness and failure strain for different lengths. (**d**) Fractured model for different lengths shows a similar failure mechanism.

**Figure A2:**
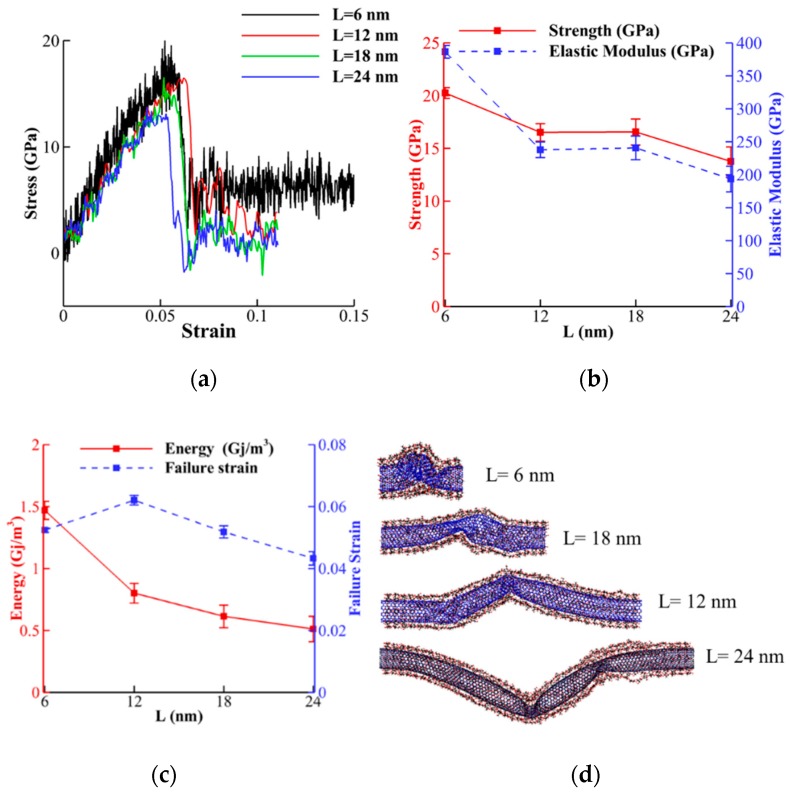
Effect of length on the compressive properties of CNT-wrapped cellulose with *V_f_* = 0.55. (**a**) Stress-strain curves. (**b**) Variation of strength and Young’s modulus for length. (**c**) Variation of toughness and failure strain for different lengths. (**d**) Fractured model for different lengths shows a similar failure mechanism.

## Appendix A. The Effect of Length on the Tensile and Compressive Properties

In this section the effect of length on the tensile properties of CNT-wrapped cellulose with *V_f_* = 0.55 is evaluated. The procedure for applying tensile test is explained in Section 2.2 and here only the length of the fiber varies from 6 to 24 nm with 6 nm intervals. The stress-strain curves and extracted strength, Young’s modulus, toughness, and failure strain from stress-strain curves are shown in Figure A1a–c. The result shows that strength, toughness, and failure strain do not change significantly by increasing the length from 6 to 24 nm (less than 20%). The Young’s modulus, however, changes from 400 GPa for 6 nm to 250 GPa (35% drop) for 12 nm and remains constant for longer lengths (Figure A1b). Additionally, the failed structures for different lengths shows a similar failure mechanism of breaking cellulose chains first and then CNT break down and no significant length dependency was observed (Figure A1d).

Figure A2a–c displays the stress-strain curves and extracted strength, Young’s modulus, toughness, and failure strain from stress-strain curves. The result shows that all compressive mechanical properties, except failure strain, significantly depend on the length of the fiber. For example, both strength and Young’s modulus drop by approximately 35% by increasing the length from 6 to 24 nm. The toughness, however, has the major drop (66%) by changing from 1.5 Gj/m^3^ for 6 nm to 0.5 Gj/m^3^ for 24 nm. Similar trends for critical buckling of CNTs have been previously reported [65,66,67]. The failed structures (shown in Figure A2d) illustrate that by increasing length, instead of one, multiple flattening forms similar to previously reported failed strictures of CNTs [65,66,67].
